# Organotin(IV) Derivatives of L-Cysteine and their *in vitro* Anti-Tumor Properties

**DOI:** 10.1155/S1565363304000044

**Published:** 2004

**Authors:** Christos T. Chasapis, Sotiris K. Hadjikakou, Achilles Garoufis, Nick Hadjiliads, Thomas Bakas, Maciej Kubicki, Yang Ming

**Affiliations:** 1 Inorganic and Analytical Chemistry Department of Chemistry University of Ioannina Ioannina 45110 Greece; 2 Physics of Material Laboratory Department of Physics University of Ioannina Ioannina 45110 Greece; 3 Department of Chemistry A. Mickiewicz University, ul. Grunwaldzka 6 Poznan 60- 780 Poland; 4 National Research Laboratory of Natural and Biomimetic Drugs Peking University Beijing Beijing 100083 China

## Abstract

The synthesis and characterization of the organotin compounds [(n-C_4_H_9_)_2_Sn(cys)] (1), [(C_6_H_5_)_2_Sn(cys)] (2), [(C_6_H_5_)_3_Sn(Hcys).(H_2_o)] (3), {[(CH_3_)_2_Sn(Kcys)_2_].2(H_2_0)} (4), {[(n-C_4_H_9_)_2_Sn(Kcys)_2_].2(H_2_0)} (5) and {[(C_6_H_5_)_2_Sn(Kcys)_2_].2(H_2_0)} (6) (where H_2_cys = L-cysteine) are reported. The compounds have been characterized by elemental analysis and ^1^H-NMR, Uv-Vis, FT-IR and MOssbauer spectroscopic techniques. Attempted recrystallization of (2) in DMSO/methanol 2:1 solution yielded after several days unexpectedly the dimeric compound bis(tri-phenyltin)sulphide {[(C_6_H_5_)_3_Sn]_2_S} (7) which has been characterized by x-ray analysis. The structure of the parent complex (2) as well as the mechanism of the decomposition of cysteine are being further investigated. The in vitro anticancer activity of complexes (I)- (6), against human leukemia (HL60), human liver (Bel7402), human stomach (BGC823) and human cervix epithelial human carcinoma (Hela), nasopharyngeal carcinoma (KB) and lung cancer (PG) tumor cells, were evaluated.


### Organotin(IV) Derivatives of L-Cysteine and their *in vitro* Anti-Tumor Properties.

Christos T. Chasapis[a], Sotiris K. Hadjikakou[a,*] Achilles Garoufis[a], Nick Hadjiliadis[a,*] Thomas Bakas[b], Maciej Kubicki[c], and Yang Ming[d]

[a]*Inorganic and Analytical Chemistry, Department of Chemistry, University of Ioannina, 45110 Ioannina, Greece;*

[b]*Physics of Material Laboratory, Department of Physics, University of Ioannina, 45110 Ioannina, Greece.*

[c] *Department of Chemistry, A. Mickiewicz, University, ul. Grunwaldzka 6, 60-780 Poznan, Poland..*

[e] *National Research Laboratory of Natural and Biomimetic Drugs, Peking University, Beijing 100083, Beijing, P. R. China.*

### ABSTRACT

The synthesis and characterization of the organotin compounds $[(n\text{-}C_4H_9)_2Sn(cys)]$ (**1**), $[(C_6H_5)_2Sn(cys)]$ (**2**), $[(C_6H_5)_3Sn(Hcys){\cdot}(H_2O)]$ (**3**), $\{[(CH_3)_2Sn(Kcys)_2]{\cdot}2(H_2O)\}$ (**4**), $\{[(n\text{-}C_4H_9)_2Sn(Kcys)_2]{\cdot}2(H_2O)\}$ (**5**) and $\{[(C_6H_5)_2Sn(Kcys)_2]{\cdot}2(H_2O)\}$ (**6**) (where $H_2cys$= L-cysteine) are reported. The compounds have been characterized by elemental analysis and $^1$H-NMR, Uv-Vis, FT-IR and Mössbauer spectroscopic techniques. Attempted recrystallization of (**2**) in DMSO/methanol 2:1 solution yielded after several days unexpectedly the dimeric compound bis(tri-phenyltin)sulphide $\{[(C_6H_5)_3Sn]_2S\}$ (**7**) which has been characterized by x-ray analysis. The structure of the parent complex (**2**) as well as the mechanism of the decomposition of cysteine are being further investigated. The *in vitro* anticancer activity of complexes (**1**) – (**6**), against human leukemia (HL60), human liver (Bel7402), human stomach (BGC823) and human cervix epithelial human carcinoma (Hela), nasopharyngeal carcinoma (KB) and lung cancer (PG) tumor cells, were evaluated.

**Keywords:** Bioinorganic chemistry, organotin(IV) compounds, mercapto amino acids, L-cysteine, anti-tumour compounds

---

*All correspondence should be addressed to:

Assistant Professor S.K. Hadjikakou; e-mail: shadjika@cc.uoi.gr; tel. +30-26510-98374

Professor N. Hadjiliadis: e-mail: nhadjil@cc.uoi.gr, tel. +30-26510-98420; fax +30-26510-44831

### 1. INTRODUCTION

The biological activity of organotin(IV) compounds is well known /1-3/. Most organotin(IV) compounds are generally toxic /3/. The binding of organotins by thiol groups, on the other hand, is significant in biological systems /4/. A great deal of work has been reported thus far, on the interaction of organotin compounds with thiol-containing mercapto-amino acids such as cysteine and its derivatives /3, 5-14/. The interpretation of the results however, was rather controversial. Thus, based on Mössbauer spectroscopy Barbieri *et al.* /6/ proposed a penta-coordinate structure for the L-cysteinato(S-)-triorgano-tin(IV) hydrate [$R_3Sn(Cys)(H_2O)$] (R= Me- or Et- group) /6, 8/. A penta-coordinate geometry around tin atom was also proposed by Huger *et al.* /10/ for [$Ph_2Sn(Cys)$]. The anti-leukemic activity of compound [$Ph_2Sn(Cys)$] was evaluated by Huber *et al.* /8/ and was found to be remarkable in relation to its low toxicity. A tetrahedral arrangement around tin(IV) was proposed for [$Ph_3Sn(HCys)$] by Hyams *et al.* /14/ and Huger *et al.* /10/. According to spectroscopic data Bamgboye *et al.* /7/ proposed a polymeric chain structure for [$(Ph_3Sn)_2$cys] with penta-coordinated tin atom /7/, while a double anionic structure was proposed by Domazetis *et al.* /13/ for the {[$(Me)_2Sn(OH)_2(cys)]^{2-}$} at high pH. The crystal structure of the ethyl-L-cyteinatoS,N-(chloro-dimethyl) stannate(IV) [$(Me)_2Sn(Cl)(SCH_2CH(NH_2)$ $COOC_2H_5$] /11/ was determined by X-ray analysis and indicated S, N chelated coordination of the ethyl-ester of cysteine with the tin atom in a distorted trigonal bibyramidal configuration.

Another demand for such studies raised from the potential pharmaceutical application of organotin compounds /5, 15/. Today, a number of organotin (IV) derivatives are known to have an efficient anti-tumour activity /15/. A comparison of the structures of the active and inactive compounds suggests that all active compounds should conform to the following requirements: (i) to have available coordination positions around Sn, (ii) to have a relatively stable ligand-Sn bond (e.g. Sn-N and Sn-S) with low hydrolytic decomposition /5/.

The aim of the present work is the exploration of new synthetic routes for the synthesis of organotin(IV) complexes with cysteine, the better characterization of the compounds formed and the evaluation of anti-cancer activity of the complexes derived and characterized.

### 2. EXPERIMENTAL

### Materials and Instruments:

All solvents used were reagent grade, while organotin chlorides (Aldrich), cysteine (Merck) were used with no further purification. Elemental analysis for C, H, N, and S were carried out with a Carlo Erba EA Model 1108. Infra-red spectra in the region of 4000-370 $cm^{-1}$ were obtained in KBr discs while far-infra-red spectra in the region of 400-50 $cm^{-1}$ were obtained in polyethylene discs, with a Perkin-Elmer Spectrum GX FT-IR spectrometer. A Jasco Uv/Vis/NIR V 570 series spectrophotometer was used to obtain the electronic absorption spectra.. The $^{1}H$-NMR spectra were recorded on a Bruker AC250 MHFT-NMR instrument in $CDCl_3$ solutions. Chemical shifts δ are given in ppm using TMS as an internal reference. The $^{119}Sn$

Mössbauer spectra were collected at various sample temperatures, with a constant acceleration spectrometer equipped with $CaSnO_3$ source kept at room temperature.

*Synthesis of L-cysteinato-k$^2$S,O-di-n-butyl-tin(IV), [(n-$C_4H_9$)$_2$Sn(cys)]} (1):*

Cysteine ($H_2$cys) (0.121 g, 1.0 mmol) was treated with $Na_2CO_3$ (0.106 g, 1.0 mmol) $Na_2CO_3$ in $H_2O$ (17 cm$^3$) followed by addition of a solution of di-butyltin(IV) dichloride [(*n*-$C_4H_9$)$_2$SnCl$_2$] (0.152 g, 0.5 mmol) in methanol (3 cm$^3$). Chloroform (20 cm$^3$) was then added to this solution and the resulting mixture was stirred for 3 h. The organic layer was dried over $Na_2SO_4$ and filtered to yield a clear solution. Removal of solvent yielded a white powder of compound (**1**). Yield 0.045 g (26%); m.p. = 208-210 °C;. Elemental analysis, found: C: 37.63; H: 6.76; N: 3.57, S: 8.96 %, calculated for $C_{11}H_{23}NO_2SSn$: C: 37.53; H: 6.58; N: 3.98, S: 9.11 %. IR (cm$^{-1}$): 3240s, 2956vs, 2923vs, 2855s, 1641vs, 1545m, 1464m, 1429m, 1390s, 1322s, 1300, 1050m, 867m, 800w, 681m, 583m; 460s, 397m.

*Synthesis of L-cysteinato-k$^2$S,O-diphenyl-tin(IV), [($C_6H_5$)$_2$Sn(cys)]} (2):*

Cysteine ($H_2$cys) (0.243 g, 2 mmol) was treated with $Na_2CO_3$ (0.212 g, 2 mmol) in $H_2O$ (17 cm$^3$) followed by addition of a solution of di-phenyltin(IV) dichloride [($C_6H_5$)$_2$SnCl$_2$] (0.344 g, 1 mmol) in methanol (3 cm$^3$). A white precipitate of (**2**) was formed immediately. Yield 0.08 g (20 %); m.p.= 240-245 °C;. Elemental analysis, found: C: 45.86; H: 3.89; N: 3.62, S: 8.35 %, calculated for $C_{15}H_{15}NO_2SSn$: C: 45.96; H: 3.85; N: 3.57, S: 8.18 %. IR (cm$^{-1}$): 3240s, 2956vs, 2923vs, 2855s, 1641vs, 1545m, 1464m, 1429m, 1390s, 1322s, 1300m, 1050m, 867m, 800w, 681m, 583m; 460s, 396m.

Attempted re-crystallisation of (**2**) in DMSO/methanol 2:1 solution yielded, after several days, *unexpectedly*, the dimeric compound bis(tri-phenyltin)sulfide {[($C_6H_5$)$_3$Sn]$_2$S} (7) /16/.

*Synthesis of L-cysteinato(S-)-triphenyl-tin(IV) hydrate, [($C_6H_5$)$_3$Sn(Hcys)($H_2O$)]} (3):*

Cysteine ($H_2$cys) (0.121 g , 1 mmol) was treated with ) $Na_2CO_3$ (0.106 g, 1 mmol in $H_2O$ (17 cm$^3$) and a solution of tri-phenyltin(IV) chloride [($C_6H_5$)$_2$SnCl$_2$] (0.385 g, 1 mmol) in methanol (6 cm$^3$) was added. A white precipitate of (**3**) was formed immediately. Yield 0.24 g (50 %); m.p.= 224-228 °C;. Elemental analysis, found: C: 51.90; H: 4.46; N: 3.19, S: 6.38 %, calculated for $C_{21}H_{23}NO_3SSn$: C: 51.67; H: 4.74; N: 2.87, S: 6.57 %. IR (cm$^{-1}$): 3426s, 3065m, 2925m, 2853s, 1637vs, 1429s, 1388s, 1340s, 1300m, 1074s, 728vs, 697vs, 540m; 451m, 396m.

*Synthesis of {[($CH_3$)$_2$Sn(Kcys)$_2$]·2($H_2O$)} (4), {[(n-$C_4H_9$)$_2$Sn(Kcys)$_2$]·2($H_2O$)} (5) and {[($C_6H_5$)$_2$Sn(Kcys)$_2$]·2($H_2O$)} (6):*

Cysteine ($H_2$cys) (0.121 g, 1 mmol) in $H_2O$ (5 cm$^3$) was treated with solution of KOH 1N (2 cm$^3$, 2 mmol). The resulting solution was concentrated to a small volume, where upon a colorless oily product was formed. The oil was then dissolved in methanol (10 ml) followed by addition of a solution of the appropriate di-organotin(IV) dichloride (0.5 mmol), (0.109 g di-methyl(IV) dichloride, 0.152 g, di-butyltin(IV) dichloride, 0.170 g di-phenyltin(IV) dichloride) in methanol (5 ml). The solution was stirred for 1 h and a white precipitate of KCl was formed. The mixture was then filtered off and the resulting clear solution was concentrated to dryness in a rotary evaporator under vacuum to yield a white powdered product.

{[$(CH_3)_2Sn(Kcys)_2]\cdot 2(H_2O)$} (4): Yield 0.053 g; m.p.= 178-185 °C;. Elemental analysis, found: 18.91; H: 3.69; N: 5.65, S: 12.25 %, calculated for $C_8H_{20}K_2N_2O_6S_2Sn$: C: 19.17; H: 4.02; N: 5.59, S: 12.79 %. IR ($cm^{-1}$): 3447s, 2925m, 1637vs, 1499m, 1399m, 1341m,1298m, 1124m, 853m, 418s, 397m.

{[$(n\text{-}C_4H_9)_2Sn(OH)_2(cys)_2]K_2$} (5): Yield 0.057 g; m.p.= 159-165 °C;. Elemental analysis, found: C: 29.08; H: 5.82; N: 4.22, S: 10.75 %, calculated for $C_{14}H_{32}K_2N_2O_6S_2Sn$: C: 28.73; H: 5.50; N: 4.79, S: 10.95 %. IR ($cm^{-1}$): 3448s, 2957m, 2924m, 2854m, 1618vs, 1509m, 1459m, 1425m,1396m, 1341m, 1293m, 1141m, 1053m, 847m, 533m, 397m.

{[$(C_6H_5)_2Sn(OH)_2(cys)_2]K_2$} (6): Yield 0.06 g; m.p.= 219-222 °C;. Elemental analysis, found: C: 34.75; H: 3.60; N: 4.67, S: 10.38 %, calculated for $C_{18}H_{24}N_2O_6S_2Sn$: C: 34.57; H: 3.86; N: 4.48, S: 10.25 %. IR ($cm^{-1}$): 3435s, 2924m, 1625vs, 1509m, 1428m, 1388m, 1336m, 1310m, 1112m, 852m, 732m, 697m;538m, 455m, 418m, 397m).

*Biological test*

Anticancer activity against Human leukemia (HL60), Human liver (Bel7402), Human stomach (BGC823) and Human cervix epithelial human carcinoma (Hela), Nasopharyngeal Carcinoma (KB), Lung Cancer (PG) tumor cells, *in vitro* was evaluated by using a system based on MTT. Tumor cells were grown in RPMI1640 medium supplemented with 10% freshly inactivated fetal calf serum (FCS) and antibiotics from the cells harvested from exponential phase were seeded equivalently onto a 96-well plate, the compounds studied were added in a concentration gradient, and the final concentrations were maintained at 100, 10, 1, and 0.1 μm, respectively. The plate was kept at 37 °C in a humidified atmosphere of 5% $CO_2$ and incubated for 48 h. MTT solution was added to each well. After incubation for 4 h at 37 °C, acid-isopropanol was added to all wells. The measurements of absorbance of the solutions related to the number of living cells were carried out on a Bio-Rad Model 450 Microplate Reader at 570 nm.

*Crystal structure of bis(triphenyl-tin(IV))sulphide {$[(C_6H_5)_3Sn]_2S$} (7):*

The crystal structure of compound (7), ($C_{36}H_{30}SSn_2$) was solved with SHELXS97 [18] and refined by full-matrix least-squares procedures on $F^2$. Data were collected on a KUMA KM4CCD four-circle diffractometer /18/, with a CCD detector. (Compound (7): ($C_{36}H_{30}SSn_2$), MW= 732.04, orthorhombic, space group $P2_12_12_1$, a= 9.8419(8) Å, b= 17.6680(13) Å, c= 18.4958(14), Z= 4, V= 3216.2(2) $Å^3$, Z= 4, T= 293(2) K, ρ(cald)= 1.512 g $cm^{-3}$, μ= 1.640 $mm^{-1}$, Crystal size: 0.3 x 0.1 x 0.1 $mm^3$, goodness-of-fit on $F^2$= 1.113, Final R indices [I>2.5sigma(I)]; R1 = 0.0501, wR2 = 0.0442).

### 3. RESULTS AND DISCUSSION

### General aspects

Compound (**1**) has been synthesized using a water/methanol/chloroform 17:3:20, solvent system while compounds (**2**) and (**3**) were synthesized in a water/methanol 17:3 according to equation (1)

$$R_nSnCl_{4-n} + 2\ H_2Cys + (4-n)\ Na_2CO_3 \longrightarrow$$

$$R_nSn(H_{n-2}Cys) + 2\ (4-n)\ NaCl + (4-n)\ H_2O\ + (4-n)\ CO_2 \quad \text{(eq 1.)}$$

(where n=2 and R= $C_4H_9$-(**1**), $C_6H_5$- (**2**) or n=3 and R= $C_6H_5$- (**3**)).

Compounds (**4**), (**5**), (**6**) have been synthesized in a water/methanol 7:10 solution, according to equation (2)

$$R_2SnCl_2 + 2\ H_2Cys + 4\ KOH \longrightarrow \{[R_2Sn\,(K\,Cys)_2] \bullet 2(H_2O)\}\} + 2\ KCl + 2\ H_2O \quad \text{(eq. 2)}$$

where R=$CH_3$-(**4**), $C_4H_9$-(**5**), $C_6H_5$-(**6**)

The formulae of complexes synthesized were deduced from elemental analysis and spectroscopic data. Complexes (**1**) - (**3**) are soluble in DMSO, while complex (**1**) is also soluble in chloroform, dichloromethane and acetone, (**3**) is soluble in methanol and acetone, while (**4**)-(**5**) are soluble in methanol.

Compound (**3**) was synthesized previously from {$(C_6H_5)_3Sn(OH)$} and L-cysteine in chloroform/water or methanol/ether media by Huber *et al.* /10/ and Hyams *et al.* /14/. Huber *et al.* /8, 10/ have also, reported the synthesis of {$(C_6H_5)_2Sn(cys)$} (**2**) by reacting {$(C_6H_5)_2Sn(OH)_2$} with cysteine in chloroform/water. The interaction of L-cysteine with tri-phenyltin(IV) producing {bis-(triphenyltin)cysteine} {$[(C_6H_5)_3Sn]_2(Hcys)$} was also studied by Bamgboye *et al.* /7/.

Attempted recrystallization of (**2**) in DMSO/methanol 2:1 solution yielded after several days the dimeric compound bis(tri-phenyltin) sulphide {$[(C_6H_5)_3Sn]_2S$} (**7**) which was characterized by x-ray analysis and found identical to the one previously reported by Glidewell *et al.* /16a/, Cox /16b/ and D'yachenko /16c/. The method of preparation of the crystal was different however. In addition Domazetis *et al.* /17/ detected {$[(C_6H_5)_3Sn]_2S$} by mass spectroscopic techniques.

The de-sulphuration reaction of cysteine is known to play a role in thiol catabolism in biological systems /19/.

A possible mechanism of de-sulphuration of {$(C_6H_5)_2Sn(cys)$} (**2**) may be summarized below, knowing that such compounds decompose slowly on standing, forming bis(tri-phenyltin) sulphide {$[(C_6H_5)_3Sn]_2S$} /17/. Cysteine was also detected by IR and mp in the precipitate.

$$(C_6H_5)_2Sn(cys) \xrightarrow[H_2O]{DMSO\,/\,MeOH} ((C_6H_5)_3Sn)_2S + 2Sn + 2\,cystine + alanine$$

### Spectroscopy:

*Infra-red*: Characteristic frequencies in IR spectra of complexes (1)-(6) are listed in Table 1.

**Table 1.**

Characteristic vibration bands ($cm^{-1}$) in infra-red spectra of complexes or ligands

| | $\nu_\alpha NH_3^+$ | $\nu_\alpha(CH_2)$ | $\nu$ (S-H) | $\nu_\alpha(COO^-)$ | $\nu_s(COO^-)$ | $\nu$(C-S) | $\nu$(Sn-S) | $\nu$(Sn-O) | ref |
|---|---|---|---|---|---|---|---|---|---|
| L-Cys | 3428<br>3166 | 2994 | 2551 | 1575 | 1395 | 691 | | | /13,<br>22/ |
| (1) | 3306<br>3240 | 2956 | absent | 1637 | 1390 | 681 | 397 | 583 | |
| (2) | 3449<br>3269 | 2919 | absent | 1611 | 1380 | 659 | 396 | 570 | |
| (3) | 3436<br>3052 | 2925 | absent | 1627 | 1388 | 672 | 395 | 540 | |
| (4) | 3447 | 2924 | absent | 1637 | 1399 | 675 | 397 | 528 | |
| (5) | 3431 | 2949 | absent | 1618 | 1395 | 677 | 392 | 533 | |
| (6) | 3435<br>3042 | 2924 | | 1625 | 1388 | 589 | 374 | 538 | |

$\nu_\alpha$= antisymmetric strain, $\delta_\alpha$= antisymmetric bend.

Both coordination and increase in the ionic character of the carboxylate group tend to reduce the asymmetric $\nu$(O-C=O) frequency /12, 20-21/. The $\nu_{as}$(COO) is observed at 1575 $cm^{-1}$ in free cysteine /22/. The corresponding $\nu_{as}$(COO) vibration in the spectra of complexes (1)-(3) shift to higher frequencies (Table 1) indicating coordination of cysteine through the carboxyl group to tin atom /10, 13/. The magnitudes of the [$\nu_{as}$(COO) - $\nu_s$(COO)] ($\Delta\nu$) separation in complexes (1)-(3) ($\Delta\nu$=239±8) are comparable to those reported for organotin derivatives of L-cysteine and L-cysteine ethyl ester /12, 13, 21/. The conclusions drawn above are further supported by the presence in the IR spectra of a sharp band at 560±20 $cm^{-1}$, which was assigned to the Sn-O stretching vibration /21/ (Table 1). The organotin compounds (4)-(6) have carbonyl $\nu_{as}$(COO) vibration at 1627±10 $cm^{-1}$. The band position of $\Delta\nu$ [$\nu_{as}$(COO) -$\nu_s$(COO) =228±10] in the case of compounds (4)-(6) indicate coordination of cysteine through the carboxyl group to tin atom as well /12, 13, 21/. The stretching frequencies for $NH_2$ group in the ir spectra of compounds (1)-(6) can help to distinguish coordinated from free amino groups. The $\nu(NH_2)$ of the free amino group is observed at 3166$cm^{-1}$ in cysteine /13, 22/. The assignment of the $-NH_2$ group has been made by deuterization of the samples using $D_2O$. The corresponding $\nu(NH_2)$ vibrations of the organotin derivatives (1)-(6) remain at the same frequency indicating that coordination through the amino group of cysteine to tin atoms does not take place. These conclusions are further supported by the absence in the far-IR of a band assignable to $\nu$(Sn-N) /21/. The absence of $\nu$(S-H) bands in the IR spectra of compounds (1)-(6) shows a coordination of sulfur to tin atom. The stretching frequency of C-S bond in IR spectra of cysteine is observed at 691 $cm^{-1}$ /13, 22/. The corresponding vibration v(C-S) of organotin derivatives (**1**)-(**6**) shifts to lower frequencies (Table 1) supporting the same conclusion.

*$^{119}Sn$ Mössbauer spectra*: Mössbauer parameters of compounds (**1**)-(**6**) are listed in Table 2, while the quality of the $^{119}$Sn Mössbauer spectrum of the compounds measured at 85K are shown in Figure 1.

**Table 2**

Mössbauer parameters of compounds (**1**)-(**6**)

| Compound | δ ($mms^{-1}$) | ΔEq ($mms^{-1}$) | Area % | δ ($mms^{-1}$) | ΔEq ($mms^{-1}$) | Area% |
|---|---|---|---|---|---|---|
| **1** | 1.25 | 2.77 | 68 | 1.51 | 2.94 | 32 |
| **2** | 1.32 | 2.86 | 71 | 1.21 | 2.65 | 29 |
| **3** | 1.31 | 1.44 | 78 | 1.27 | 2.96 | 22 |
| **4** | 1.62 | 2.75 | 42 | 1.16 | 2.82 | 58 |
| **5** | 1.42 | 1.77 | 37 | 1.41 | 3.02 | 63 |
| **6** | 1.42 | 2.40 | 45 | 1.15 | 2.35 | 55 |

The occurrence of two symmetric Lorentzian doublets in the spectra of compounds (**1**)-(**6**) show two distinct coordination sites with tin /2, 23/. The values between the quadruple splitting resonances of the two symmetric Lorentzian doublets in the spectrum of compound (**3**) (Table 2) differ significantly, allowing the assignment of the environment around the tin atom /7/ to be made. In the case of compound (**3**), a resonance with smaller splitting (tin moiety with contribution of 80% in the sample) is similar to that in $[(Ph_3Sn)_2Cys]$ /7/ and is assigned to a tetrahedral $[Ph_3SnS]$ environment, while that with the higher splitting resonance corresponds to the penta-coordinated $Ph_3Sn(Cys,S\text{-})(OH_2)$ environment /7/. For compound (**5**), the inner doublet was assigned to the BuSn-S moiety while the outer doublet was assigned to the BuSn-O moiety /12/.

The quadruple splitting values (ΔEq) of complexes (**1**)-(**6**) are typical of organotin thiolate derivatives /12/. The ΔEq values of compounds (**1**), (**2**), (**4**) and (**6**) are in the range of 2.35 to 3.02 $mms^{-1}$ showing octahedral arrangement around tin(IV) /7, 14, 24/. The low values of the quadruple splitting parameter, ΔEq ($\leq$1.77 $mms^{-1}$), in the case of complexes (**3**) and (**5**) are characteristic of a tetrahedral geometry around tin /25/.

**Figure 1.** The $^{119}$Sn Mössbauer spectra of compounds (**1**)- (**6**).

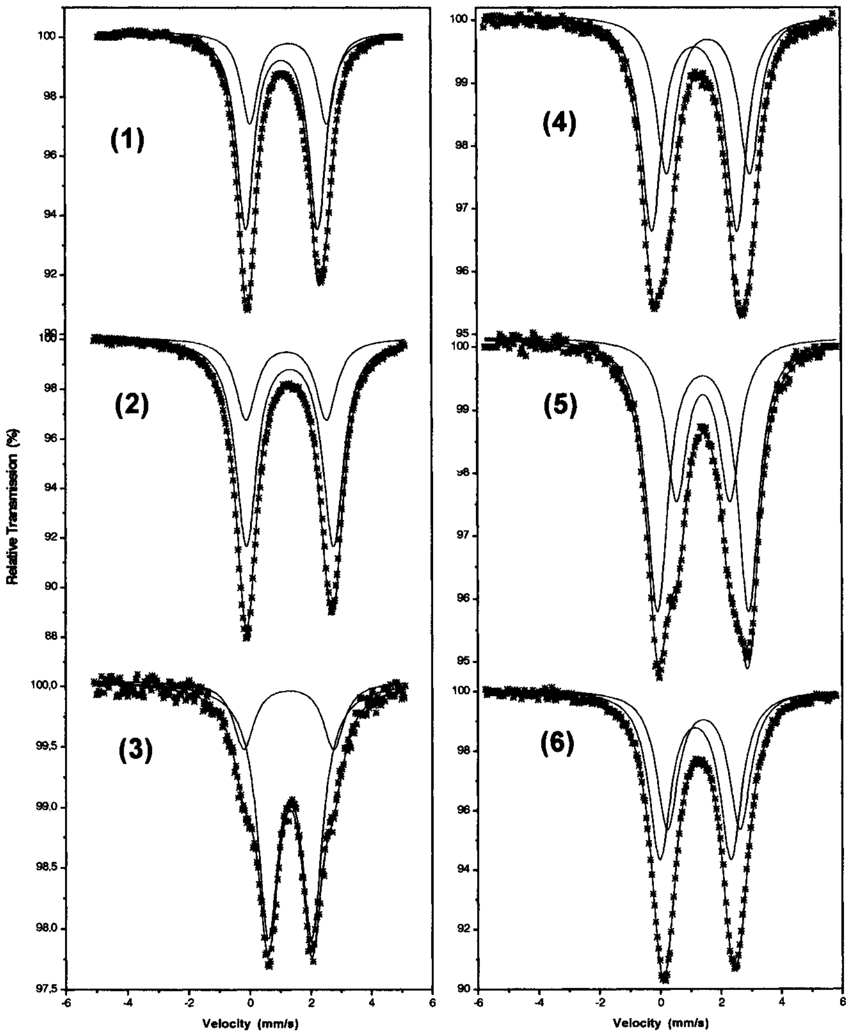

*NMR spectra:* Chemical shifts observed in $^{1}$H-NMR of L-cysteine and its compounds (**1**), (**2**), (**4**) and (**5**) are listed in Table 3.

$^{1}$H-NMR spectrum of free cysteine in *DMSO-d$_6$* shows signals at 2.86 and 3.12 ppm for -$CH_2$- and -CH- protons respectively. The signal in $^{1}$H-NMR spectrum of complex (**2**) in *DMSO-d$_6$* at 2.91 ppm is assigned to the -$CH_2$- and -CH- protons of coordinated cysteine.

The corresponding resonance signals of -$CH_2$- and -CH- groups in $^{1}$H-NMR of free cysteine in $CD_3OH$ are observed at 3.10 and 4.02 ppm respectively; these are shifted to 2.94-2.98 ppm and 3.7 ppm respectively in the case of (**4**) and at 2.89-2.98 ppm and 3.6 ppm in the case of complex (**5**), indicating coordination of cysteine to tin(IV) atom.

**Table 3**

$^1H$ NMR chemicals shifts of the compounds in ppm.

| Compound | Chemical Shifts of Organotin Moiety | Shifts of L-cysteine | |
|---|---|---|---|
| | | $-CH_2$ | -CH |
| Cysteine $CD_3OD$ | | 3.10 | 4.02 |
| Cysteine DMSO $d_6$ | | 2.86 | 3.12 |
| (1) $CDCl_3$ | 0.88-1.56 ($n$-$C_4H_9$-) | 2.67-3.04 | 3.48 |
| (2)DMSO-$d_6$ | 7.25-7.88 ($C_6H_5$-) | | 2.91 |
| (4) $CD_3OD$ | 0.81 ($CH_3$-) | 2.94-2.96 | 3.7 |
| (5) $CD_3OD$ | 0.87-1.72 ($n$-$C_4H_9$-) | 2.89-2.98 | 3.6 |

$^1$H-NMR spectrum of complex (**1**) in $CDCl_3$ shows signals for the $-CH_2-$ and -CH- protons of cysteine at 2.67-3.04 and 3.48 ppm.

The $^{13}$C-NMR spectrum of free cysteine in DMSO-$d_6$ shows signals at 169.86 ppm for the $-COO^-$ and at 56.50 ppm, 26.12 ppm for the Ca and Cb atoms respectively. The $^{13}$C-NMR spectrum of the complex (**1**) shows resonance signals at 174.34 ppm for the $-COO^-$ and at 57.25, 31.14 ppm for the Ca and Cb atoms respectively. The signals of the carboxylic $-COO^-$ and Cb atoms have been shifted downfield by 4.5 and 5.0 ppm respectively towards the corresponding signals of free cysteine indicating the cysteinato-k$^2$S,O-coordination mode in the case of complex (**1**). The signal at 57.25 ppm is attributed to the Ca atom of the coordinated cysteine while four new signals also appear in the $^{13}$C-NMR spectrum of complex (**1**) at 28.33, 27.16 ($J$ Sn-Ca'= 51 Hz), 23.25 and 14.54 ppm which are attributed to the Cb', Ca', Cc' and Cd' atoms of the butyl group.

*Biological tests:* The *in vitro* anticancer activity of the complexes (**1**) – (**6**), against Human leukemia (HL60), Human liver (Bel7402), Human stomach (BGC823) and Human cervix epithelial human carcinoma (Hela), Nasopharyngeal Carcinoma (KB), Lung Cancer (PG) tumor cells is shown in Table 4.

Results show that complexes (**1**), (**2**) and (**5**) exhibit high cell toxicity against Hela while complexes (**1**), (**3**) and (**6**) show high cell toxicity against BGC. Cell toxicity at concentrations of 10 μM and the cells were viable to less than 10 %. A further systematic investigation of the biological properties of these metal compounds is recommended focusing on their mutual significance for tumor induction of neoplastic growth.

### ACKNOWLEDGEMENTS.

This work was carried out in partial fulfillment of the requirements for a M.Sc. thesis of Mr Christos Chasapis within the graduate program EPEAEK in Bioinorganic Chemistry financed by the Ministry of Education of Greece and coordinated by Professor N. Hadjiliadis.

**Table 4**

The *in vitro* anticancer activity of the complexes (**1**) – (**6**), against Human cervix epithelial human carcinoma (Hela), Human stomach (BGC823), Human leukemia (HL60), Lung Cancer (PG), Human liver (Bel7402) and Nasopharyngeal Carcinoma (KB) tumor cells

| Sample | Cell line | Concentration (μM) | Inhibition rate (%) | Evaluation of anti-cancer activity *in vitro.* |
|---|---|---|---|---|
| (1) | Hela | 0.1<br>1.0<br>10<br>100 | -9.43<br>27.97<br>77.12<br>106.12 | + |
| (2) | Hela | 0.1<br>1.0<br>10<br>100 | -16.73<br>0.54<br>92.70<br>95.62 | + |
| (4) | Hela | 0.1<br>1.0<br>10<br>100 | 17.66<br>15.17<br>14.24<br>64.37 | - |
| (5) | Hela | 0.1<br>1.0<br>10 | -6.98<br>-7.93<br>8.37 | - |
| (6) | Hela | 0.1<br>1.0<br>10<br>100 | -38.93<br>-38.91<br>71.98<br>96.14 | + |
| (1) | BGC | 0.1<br>1.0<br>10<br>100 | -2.80<br>34.20<br>67.61<br>84.38 | + |

**Table 4** (continued)

| | | | | |
|---|---|---|---|---|
| **(3)** | BGC | 0.1<br>1.0<br>10<br>100 | -7.46<br>7.93<br>68.78<br>86.58 | + |
| **(4)** | BGC | 0.1<br>1.0<br>10<br>100 | 8.14<br>3.37<br>-1.57<br>30.24 | - |
| **(6)** | BGC | 0.1<br>1.0<br>10<br>100 | -5.33<br>3.27<br>69.75<br>83.54 | - |
| **(5)** | HL-60 | 0.1<br>1.0<br>10 | -3.54<br>4.73<br>37.14 | |
| **(5)** | PG | 0.1<br>1.0<br>10 | 12.71<br>13.57<br>27.18 | |
| **(5)** | Bel-7402 | 0.1<br>1.0<br>10 | 5.91<br>11.92<br>25.32 | |
| **(5)** | KB | 0.1<br>1.0<br>10 | 8.19<br>23.3<br>65.03 | |

(+) Effect on tumor in vitro, (-) No effect on tumor in vitro.

### REFERENCES

1. A. G. Davis, *Organotin Chemistry*, VCH, Verlagsgesellschaft, Weinheim, Germany, 1997.
2. P. J. Smith, *Chemistry of Tin*, Blackie Academic & Professional, an imprint of Thomson Science, London UK, 1998.
3. L. Pellerito, L. Nagy, *Coord. Chem. Rev.*, **224**, 111 (2002).
4. (a) K. Cain, M..D. Partis, D.E. Griffiths, *Biochem. J.*, **166.**, 593 (1977). (b) B.G Farrow, A.P. Dawson, *Eur. J. Biochem.*, **86**, 85 (1978).
5. M. Nath, S. Pokharia , R. Yadàv, *Coord. Chem. Rev.*, **215**, 99 (2001).
6. R. Barbieri, F. Huber, *Gazz. Chim. Italiana*, **123**, 223 (1993).
7. O.A. Bamgboye, T. Tunde Bamgboy, P.G. Harrison, *J. Organomet. Chem.*, **306**, 17 (1986).
8. F. Huber, G. Roge, L. Carl, G. Atassi, R. Barbieri, A. Silversti, E. Rivarola, G. Ruisi, F. Di-Bianca, G. Alonzo, *J. Chem. Soc. Dalton Trans.*, 523 (1985).
9. S. Calogero, G. Valle, P.A. Cusack, P.J. SmithJohn, D. Donaldson, *Inorg. Chim. Acta*, **67**, 95 (1982)
10. V.C.D. Hager, F. Huber, R. Barbieri, A. Silvestri, *Z. Anorg. Allg. Chem.*, **471**, 194 (1980).
11. G. Domazetis, M.F. Mackay, R.J. Magee, B.D. James, *Inorg. Chim. Acta*, **34**, L247 (1979).
12. G. Domazetis, R.J. Magee, B.D. James, *J. Organomet. Chem.*, **173**, 357 (1979)
13. G. Domazetis, R. J. Magee, B. D. James, *J. Organomet. Chem.*, **162**, 239 (1978).
14. P.J. Smith, R.L. Hyams, *J. Organomet. Chem.*, **171**, C29 (1979).
15. M. Gielen, *Coord. Chem. Rev.*, **151**, 41 (1996).
16. (a) C. Glidewell, D.C. Liles, *J.Organomet.Chem.*, **224**, 237 (1981). (b) M.J. Cox, E.R.T. Tiekink, *Z.Kristallogr.-New Crystal Structures*, **212**, 351 (1997) (c) O.A.D'yachenko, A.B. Zolotoi, L.O. Atovmyan, R.G. Mirskov, M.G. Voronkov, *Dokl. Akad. Nauk SSSR.*, **237**, 863 (1977)
17. G. Domazetis, R. J. Magee, B. D. James, *Inorg. Chim. Acta*, **32**, L48 (1979).
18. (a) KUMA KM-4CCD user manual, KUMA Diffraction, Wroclaw, Poland 1999. (b) CrysAlis, program for reduction of the data from KUMA CCD diffractometer, KUMA Diffraction, Wroclaw, Poland 1999. (c) G.M. Sheldrick, Programs for Crystal Structure Analysis (Release 97-2). Institut fur Anorganische Chemie der Universität, Tammanstrasse 4, D-3400 Göttingen, Germany, 1998.
19. (a) A.L. Fluharty in "*The Chemistry of Thiol Group*" edited by S. Patai, J. Willey & Sons, New York, 599 (1974); (b) A.B. Roy, P.A. Trudinger, "*The Biochemistry of inorganic Compounds of Sulphur*", Cambridge University Press, London 1970 (c) E. Kun, "*Metabolism Pathways*", edited by D.M. Greenberg, Academic Press, New York, 375 (1969).
20. B.K.K. Ho, J.J. Zuckerman, *Inorganic Chemistry*, **12**, 1552 (1973).
21. M. Nath, R. Yadav, *Bull. Chem. Soc. Jpn*, **71**, 1355 (1998).
22. H. Susi, D.M. Byler, W.V. Gerasimowicz, *J. Molecular Structure*, **102**, 63 (1983).
23. R.V. Parish *Mossbauer Spectroscopy Applied to Inorganic Chemistry*, Long, G.J. (ed.), Plenum Press, New York, 1984.
24. J.J. Zuckerman, *Advan. Organometal*, Chem, **9**, 21 (1970).
25. D.W. Allen, J.S. Brooks, R.Formstone, *J. Organometal Chem.*, **156**, 121 (1978)

